# Physics-Guided Patch Distribution Modeling for Unsupervised Pin Defect Detection of Electrical Connectors

**DOI:** 10.3390/s26144436

**Published:** 2026-07-13

**Authors:** Gang Wang, Shu Mao, Feiping Tang, Wei Hu, Jiawei Xiang

**Affiliations:** 1 College of Mechanical and Electrical Engineering, Wenzhou University, Wenzhou 325035, China; wangg@wzu.edu.cn; 2 Ningbo Zhilun Electronics Co., Ltd., Ningbo 315403, China; jerrymao@zsnow.net.cn (S.M.); petertang@zsnow.net.cn (F.T.); tonyhu@zsnow.net.cn (W.H.)

**Keywords:** electrical connector, pin defect, anomaly detection, physics-guided learning, region of interest

## Abstract

**Highlights:**

**What are the main findings?**

- A novel physics-guided patch distribution modeling (PGPDM) method is proposed, which leverages region-of-interest (ROI) masking to introduce connector structural priors and focus feature learning on pin defect regions.
- The PGPDM method works in an unsupervised manner with only normal samples for training, well adapting to industrial scenarios with scarce defective data.

**What are the implications of the main findings?**

- Integrating physical structural knowledge into anomaly detection improves the reliability of defect inspection for connector manufacturing applications.
- The proposed method reduces the dependence on defect sample collection and manual annotation, making it suitable for practical industrial inspection scenarios with limited defective data.

**Abstract:**

Electrical connectors are critical components in electronic systems, and pin-related defects may lead to poor electrical contact, signal transmission failure, and product rejection. Automated inspection of connector pins is therefore essential for ensuring manufacturing quality and reliability. However, collecting and annotating sufficient defective samples remains challenging in industrial environments, limiting the applicability of conventional supervised learning methods. This paper proposes a physics-guided Patch Distribution Modeling (PGPDM) framework for unsupervised electrical connector pin defect detection. Trained exclusively on normal samples, it embeds connector structural priors via ROI masks to filter irrelevant background and focus feature learning on pin defect zones. Multiscale deep features from defect-free samples are utilized to build statistical distributions, and anomalies are detected by measuring feature distribution deviations at inference. Experimental results on a connector pin inspection dataset demonstrate that the proposed approach effectively highlights defective regions and improves detection performance compared with conventional feature-distribution-based anomaly detection methods. The proposed framework provides an accurate, annotation-efficient, and practically deployable solution for industrial connector inspection.

## 1. Introduction

In modern electronic manufacturing, circuit connectors serve as indispensable components for signal transmission and electrical interconnection among various modules and devices. The reliability of these connectors directly affects the operational stability and service life of electronic products [1]. During manufacturing and assembly processes, defects such as missing pins, broken pins, or pin deformation may occur due to machining errors, mechanical impacts, or handling faults. Even minor pin defects can lead to poor electrical contact, signal transmission failure, and ultimately product malfunction or rejection [2,3]. As production lines continue to move toward high throughput and intelligent manufacturing, accurate and efficient automated inspection of connector pins has become a critical quality assurance task in industrial environments.

Early machine vision-based inspection systems primarily relied on supervised image classification frameworks that combined handcrafted feature extraction with conventional machine learning classifiers [4,5]. Various image descriptors, including edge features, texture statistics, shape descriptors, and frequency-domain representations, were manually designed to characterize defect patterns, while classifiers such as Support Vector Machines (SVMs), k-Nearest Neighbors (KNNs), and Random Forests were employed for defect recognition [6,7]. Compared with traditional manual visual inspection, these approaches significantly improved inspection efficiency, repeatability, and consistency, reducing the influence of operator fatigue and subjective judgment [8]. Nevertheless, their performance heavily depended on the quality of manually engineered features. Such handcrafted representations often struggled to capture complex appearance variations under changing illumination, surface conditions, and manufacturing tolerances, limiting their adaptability to diverse industrial scenarios [9].

The rapid development of deep learning has substantially advanced the field of industrial defect detection [10]. Convolutional Neural Networks (CNNs) have demonstrated remarkable capability in automatically learning hierarchical visual representations from raw images, leading to superior performance in various inspection tasks [11]. Representative architectures such as AlexNet [12], VGGNet [13], and ResNet [14] have been widely adopted for surface defect classification and localization. More recently, Vision Transformer (ViT)-based models have attracted increasing attention owing to their ability to model long-range dependencies and global contextual information, showing promising results in industrial visual inspection applications [15,16]. Despite these achievements, most supervised deep learning approaches require large-scale annotated datasets containing sufficient defective samples. In practical manufacturing environments, however, defective products occur infrequently, while collecting and annotating defect samples is both time-consuming and expensive. Furthermore, some defect types are rare, unpredictable, or continuously evolving, making comprehensive annotation nearly impossible. These challenges have motivated growing interest in unsupervised anomaly detection methods, which learn the distribution of normal samples and identify deviations from learned patterns during inference. Among them, Patch Distribution Modeling (PaDiM) has emerged as one of the most influential approaches [17]. By modeling multiscale feature distributions extracted from pretrained convolutional networks and measuring Mahalanobis distances between test features and normal feature distributions, PaDiM achieves competitive performance across a wide range of industrial anomaly detection benchmarks without requiring defect annotations.

Although existing unsupervised anomaly detection methods have achieved encouraging results, most of them primarily focus on modeling statistical discrepancies in visual feature spaces while overlooking valuable prior knowledge associated with the physical structure of industrial products [18,19]. In many manufacturing scenarios, defects do not occur randomly across the entire image but are strongly correlated with specific functional regions and structural components [20]. For connector inspection, pin defects are inherently localized within the pin array region, whereas large portions of the image correspond to background areas, connector housings, fixtures, shadows, or illumination artifacts that are irrelevant to defect assessment. When these unrelated regions are treated equally during feature modeling, they may introduce unnecessary variations and interfere with anomaly characterization [21,22]. Consequently, integrating structural and spatial priors into the anomaly detection framework has the potential to improve both detection accuracy and robustness by guiding the model toward physically meaningful regions where defects are most likely to occur [23].

To address these limitations, this paper proposes an unsupervised Physics-Guided Patch Distribution Modeling (PGPDM) method for connector pin inspection that explicitly incorporates physical prior information. The proposed unsupervised framework only needs normal samples for training, eliminating the high costs of defective data collection and labeling. Furthermore, considering the geometric arrangement and spatial distribution characteristics of connector pins, a region-of-interest (ROI) masking strategy is designed to suppress irrelevant background information and emphasize structurally critical pin regions. By integrating product-specific physical knowledge into the feature modeling process, the proposed framework enables more focused anomaly characterization and more reliable defect detection, providing a practical and cost-effective solution for industrial connector inspection applications.

The rest of this paper is organized as follows. Section 2 introduces preliminaries on connector pin defects. Section 3 details the proposed PGPDM framework. Experimental setups, quantitative results, and visualizations are presented in Section 4. Finally, Section 5 concludes the paper.

## 2. Preliminary Knowledge

Electrical connectors are fundamental components in modern electronic equipment and are widely used in communication systems, industrial control devices, automotive electronics, and consumer products. Their primary function is to provide reliable electrical and signal transmission between different modules. Since connector reliability directly affects the performance and safety of electronic systems, strict quality inspection is required during manufacturing.

**Figure 1:**
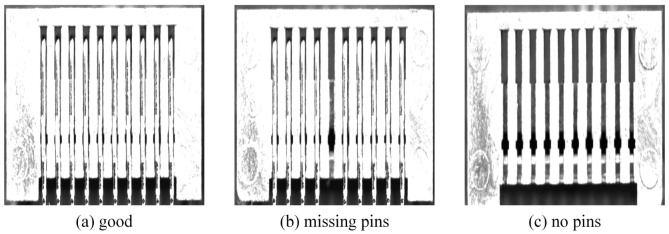
Three types of connector samples.

Among various connector defects, pin-related defects are particularly critical because pins serve as the physical interface for electrical conduction. Figure 1 illustrates the three types of connector samples considered in this study. Figure 1a shows a good connector, where all pins are present and arranged in a regular structure with uniform spacing. Figure 1b presents a connector with partial missing pins. In this case, one or several pins are absent while the remaining pins remain intact. Such defects may lead to unstable electrical contact and signal transmission failure. Figure 1c shows a connector with complete missing pins, where the entire pin array is absent. Although this defect is visually more obvious, it occurs less frequently in practical manufacturing environments.

Compared with general surface defect inspection tasks, connector pin detection exhibits several unique characteristics. First, defects are highly localized and only occupy a small portion of the image. Second, defective samples are usually scarce because production lines are designed to minimize manufacturing failures. Third, the geometric structure of connector pins is highly regular, making the inspection task strongly dependent on spatial and structural information. These characteristics make connector pin inspection a typical industrial anomaly detection problem, where obtaining sufficient defective samples for supervised training is often difficult. Consequently, unsupervised anomaly detection methods that learn normal patterns from defect-free samples provide an attractive solution for practical deployment.

## 3. Methodology

### 3.1. Overview of the Proposed Framework

**Figure 2:**
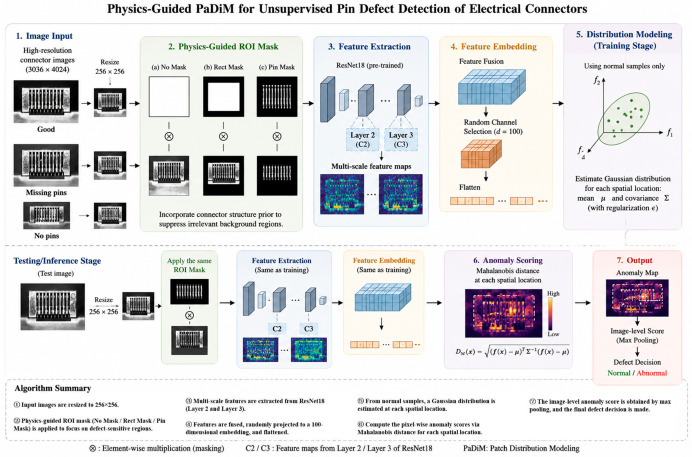
The framework of the proposed PGPDM.

The overall framework of the proposed PGPDM is illustrated in Figure 2. The method consists of two stages: a training stage and an inference stage. During training, only normal connector samples are used to construct the statistical distribution of normal features. During inference, anomaly scores are computed by measuring the deviation between test features and the learned normal distributions. Unlike the original PaDiM framework, a physics-guided region-of-interest (ROI) masking module is introduced before feature extraction. By incorporating the structural characteristics of electrical connectors, the proposed method suppresses irrelevant background regions and guides feature learning toward defect-sensitive pin areas. As a result, anomaly modeling becomes more focused on physically meaningful regions where defects are most likely to occur.

### 3.2. Physics-Guided ROI Masking

Given an input connector image

(1) $$I\in R^{H\times W\times 3}$$

where H and W denote the image height and width, respectively, a physics-guided ROI masking strategy is introduced to incorporate the structural characteristics of electrical connectors into anomaly detection. Two ROI masks are designed, namely the Rectangle Mask and the Pin Mask.

#### 3.2.1. Rectangle Mask

The Rectangle Mask is used to preserve the main connector region while suppressing irrelevant background information. Let $P_{tl}=\left(x_{1},y_{1}\right)$ and $P_{br}=\left(x_{2},y_{2}\right)$ denote the top-left and bottom-right vertices of the connector region, respectively. The rectangular mask is defined as:

(2) $$M_{r}\left(x,y\right)=\left\{\begin{array}{c} 1,\;x_{1}\leq x\leq x_{2},\;y_{1}\leq y\leq y_{2}\\ 0,\;otherwise \end{array}\right.$$

In practice, a Gaussian smoothing operation is applied to the binary mask to alleviate abrupt transitions near the ROI boundary:

(3) $$\tilde{M_{r}}=G_{\sigma_{r}}*M_{r}$$

where $G_{\sigma_{r}}$ denotes a Gaussian kernel and ∗ represents the convolution operation.

#### 3.2.2. Pin Mask

The Pin Mask further exploits the periodic spatial arrangement of connector pins. Assume that the connector contains N pins distributed uniformly along the horizontal direction. Let $c_{0}$ denote the center coordinate of the first pin, and let d represent the spacing between adjacent pins. The center coordinate of the *i*-th pin can then be expressed as:

(4) $$c_{i}=c_{0}+i\cdot d,i=0,1,\ldots ,N-1$$

For each pin, a rectangular strip with width $w_{p}$ and vertical range $\left[y_{p}^{\left(1\right)},y_{p}^{\left(2\right)}\right]$ is defined. The corresponding pin mask is given by:

(5) $$M_{p}\left(x,y\right)=\left\{\begin{array}{cc} 1, & \exists i\in \{0,\ldots ,N-1\},\left|x-c_{i}\right|\leq \frac{w_{p}}{2},\;y_{p}^{\left(1\right)}\leq y\leq y_{p}^{\left(2\right)}\\ 0, & otherwise \end{array}\right.$$

Similarly, Gaussian smoothing is applied to obtain a continuous mask:

(6) $$\tilde{M_{p}}=G_{\sigma_{p}}*M_{p}$$

Compared with the Rectangle Mask, the Pin Mask provides a stronger structural prior by explicitly emphasizing the periodically distributed pin regions where defects are most likely to occur.

#### 3.2.3. ROI-Guided Image Generation

Finally, the ROI-guided image is obtained through element-wise multiplication:

(7) $$I_{m}=I⊙M$$

where M represents either $\tilde{M_{r}}$ or $\tilde{M_{p}}$, and ⊙ denotes the Hadamard product. By suppressing irrelevant background regions and highlighting defect-sensitive structures, the proposed ROI masks guide the subsequent anomaly detection model toward physically meaningful regions.

### 3.3. Multi-Scale Feature Extraction

The ROI-filtered image is fed into a pretrained ResNet18 backbone for feature extraction. We extract feature maps from Layer 2 and Layer 3 of ResNet18:

(8) $$F_{2}\in R^{C_{2}\times H_{2}\times W_{2}}$$

(9) $$F_{3}\in R^{C_{3}\times H_{3}\times W_{3}}$$

where $C_{2}$ and $C_{3}$ represent the number of feature channels for the two feature maps, respectively. The lower-resolution feature map is upsampled to match the spatial resolution of the other feature map, and then concatenated along the channel dimension:

(10) $$F=\mathrm{Concat}\left(F_{2},F_{3}\right)$$

The fused output serves as the multi-scale feature representation containing both local texture information and high-level semantic information.

### 3.4. Feature Embedding and Distribution Modeling

The concatenated feature tensor contains redundant channels and increases computational cost. We adopt random channel selection for dimensionality reduction. Let the fused feature map be:

(11) $$F\in R^{C\times H\times W}$$

We randomly select d=100 channels to obtain the reduced feature:

(12) $$F_{r}\in R^{d\times H\times W}$$

where d ≪ C. For each spatial position $\left(x,y\right)$, we collect all feature vectors from normal training samples to form a feature set:

(13) $$X_{\left(x,y\right)}=\{f_{1},f_{2},\ldots ,f_{N}\}$$

where N denotes the total number of training samples. Assuming normal features follow a multivariate Gaussian distribution, the mean vector is calculated as:

(14) $$\mu_{\left(x,y\right)}=\frac{1}{N}\sum_{i=1}^{N}f_{i}$$

The covariance matrix with a regularization term is defined as:

(15) $$\Sigma_{\left(x,y\right)}=\frac{1}{N-1}\sum_{i=1}^{N}\left(f_{i}-\mu_{\left(x,y\right)}\right){\left(f_{i}-\mu_{\left(x,y\right)}\right)}^{T}+\varepsilon I$$

The term εI is a regularization term to guarantee numerical stability during matrix inversion, where I is the identity matrix and ε is a small scalar. Each spatial position is finally modeled as a Gaussian distribution: $N\left(\mu_{\left(x,y\right)},\Sigma_{\left(x,y\right)}\right)$.

### 3.5. Anomaly Scoring

During inference, test images go through the same preprocessing, ROI masking, and feature extraction pipelines. For the feature vector $f\left(x,y\right)$ at spatial location $\left(x,y\right)$, the anomaly score is calculated by the Mahalanobis distance:

(16) $$D_{M}\left(x,y\right)=\sqrt{{\left(f\left(x,y\right)-\mu_{\left(x,y\right)}\right)}^{T}\sum_{\left(x,y\right)}^{-1}\left(f\left(x,y\right)-\mu_{\left(x,y\right)}\right)}$$

The pixel-level anomaly map is constructed as:

(17) $$S=\left[D_{M}\left(x,y\right)\right]$$

A larger Mahalanobis distance means a larger deviation from the normal distribution, which corresponds to a higher anomaly probability. Finally, global max pooling is applied to obtain the image-level anomaly score:

(18) $$S_{\mathrm{img}}=\underset{\left(x,y\right)}{\max}S\left(x,y\right)$$

The image is classified as normal or defective according to the image-level score, and the anomaly map realizes pixel-level localization of defect regions.

## 4. Experimental Section

### 4.1. Experimental Setting

#### 4.1.1. Pin Defect Dataset Description

Experiments were conducted on an industrial electrical connector inspection dataset containing three categories: normal connectors (good), connectors with missing pins (missing pins), and connectors without pins (no pins). The dataset consists of 1048 normal samples, 192 missing-pin samples, and 12 no-pin samples, resulting in a total of 1252 images.

To construct the anomaly detection framework, only normal samples were used for model training, while both normal and defective samples were included in the testing stage. Specifically, the first 800 images were used as the training set, and the remaining 452 images were used for evaluation. Such a setting follows the practical deployment scenario of unsupervised anomaly detection, where defective samples are unavailable or extremely limited during model development.

**Figure 3:**
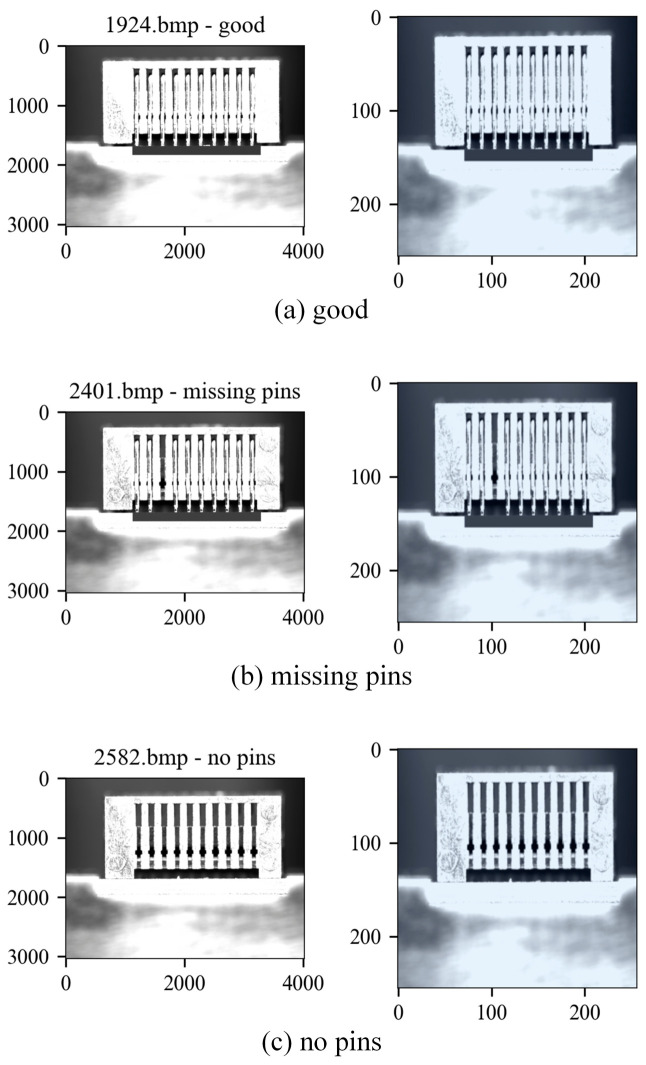
Image preprocessing of the three connector categories.

The original connector images were captured at a resolution of 4024 × 3036 pixels. This high resolution is merely a characteristic of our hardware, and the proposed method does not strictly require such high native resolution before reduction. For computational efficiency, all images were resized to 256 × 256 pixels before feature extraction. Figure 3 presents representative examples of the three categories before and after image resizing. Although the image resolution is significantly reduced, the geometric characteristics of connector pins remain clearly distinguishable, providing sufficient visual information for subsequent defect detection.

#### 4.1.2. Parameter Settings

**Figure 4:**
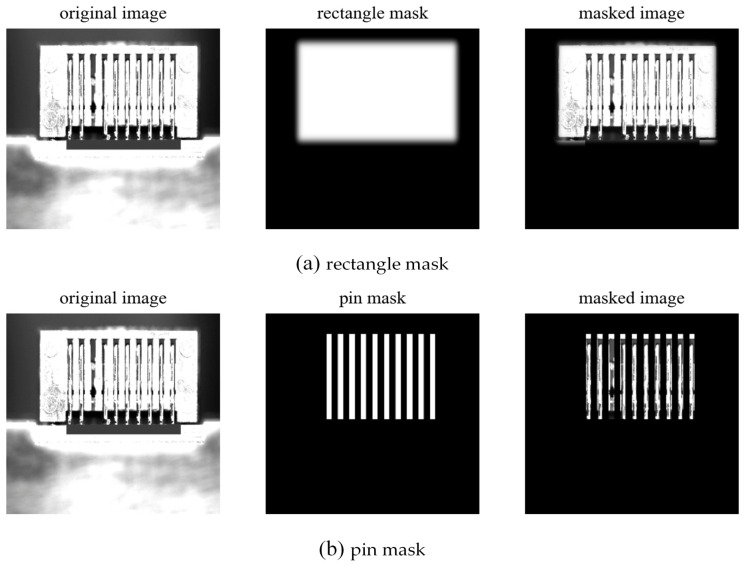
Examples of the two ROI masks.

To investigate the effectiveness of incorporating physical prior knowledge into unsupervised anomaly detection, two different region-of-interest (ROI) strategies were designed and evaluated. (1) Rectangle Mask: a rectangular ROI covering the main connector region; (2) Pin Mask: a structure-aware ROI focusing specifically on the pin array region. The Rectangle Mask aims to eliminate large irrelevant background areas while preserving the complete connector structure. In contrast, the Pin Mask further exploits the spatial distribution characteristics of connector pins and restricts feature extraction to defect-sensitive regions. Figure 4 illustrates the two ROI configurations.

For all experiments, the PGPDM framework was implemented using ResNet18 as the feature extraction backbone. Following the standard PGPDM configuration, feature maps from the second and third residual stages were extracted and fused for anomaly modeling. The dimensionality of the embedded feature representation was reduced to 100 using random channel selection. The covariance regularization parameter ε was set to 0.04 to ensure numerical stability during Mahalanobis distance computation.

The input image size was fixed at 256 × 256 pixels, and the batch size was set to 32. All experiments were conducted on a workstation equipped with an NVIDIA RTX 5070 Ti Laptop GPU using Pytorch 2.9.1. To account for the randomness introduced by feature dimension selection in PGPDM, each experiment was independently repeated five times, and the mean and standard deviation of the AUROC values were reported.

### 4.2. Experimental Results and Discussion

#### 4.2.1. Experimental Results

**Figure 5:**
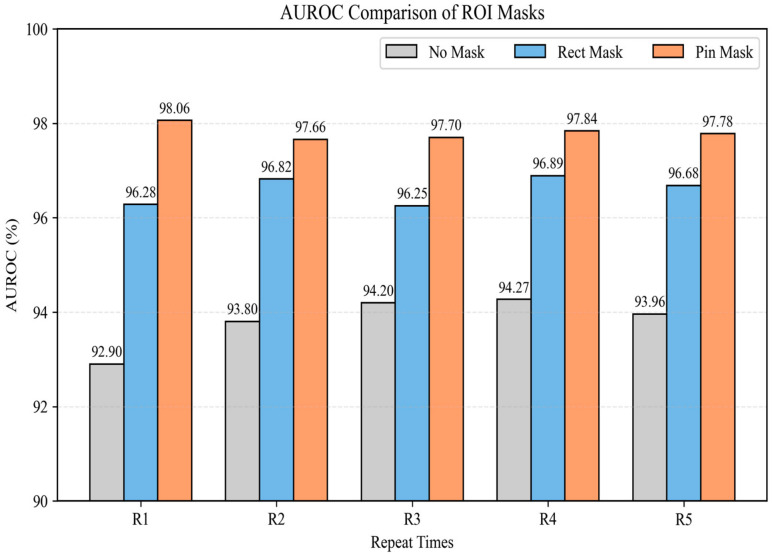
Recognition AUROC (%) of PGPDM with different ROI masks.

Table 1 and Figure 5 summarize the image-level AUROC results obtained using different ROI masking strategies across five independent runs. To better reflect the true miss-detection level, F1-Score, Precision, and Recall are introduced in Table 2.

The PGPDM framework without ROI guidance achieved an average AUROC of 93.83%, indicating that the pretrained feature distributions were capable of distinguishing defective samples from normal connectors. Notably, this “No Mask” configuration explicitly represents the standard, classical PaDiM framework, which serves as a widely recognized mainstream baseline algorithm in industrial unsupervised anomaly detection. However, the performance exhibited relatively large fluctuations across repeated runs, resulting in a standard deviation of 0.55%. As indicated in Table 2, this baseline framework yields an F1-Score of 91.17% and a Recall of 87.27%, revealing a visible miss-detection level due to its susceptibility to background clutter.

After introducing the Rectangle Mask, the average AUROC increased to 96.58%, corresponding to an improvement of 2.75 percentage points over the baseline. At the same time, the standard deviation decreased from 0.55% to 0.30%, suggesting that suppressing irrelevant background information not only improves detection accuracy but also enhances model stability. This observation confirms that background regions contribute little to defect characterization and may even introduce unnecessary feature variations during statistical distribution modeling.

The best performance was achieved by the proposed Pin Mask strategy, which obtained an average AUROC of 97.81% and a standard deviation of only 0.16%. Compared to the mainstream PaDiM baseline (No Mask), the average detection accuracy improved by 3.98 percentage points. Moreover, the reduced variance across repeated experiments indicates that the model becomes substantially more robust when guided by connector-specific structural priors. More importantly, Table 2 clearly shows that the Pin Mask strategy achieves the highest F1-Score of 93.85% and a Precision of 98.39%. Crucially, the Recall significantly rises to 89.71%, which corresponds to a prominent drop in the true miss-detection rate (from 12.73% down to 10.29%), proving its capability to capture fine-grained pin defects.

These quantitative results demonstrate that incorporating physical prior knowledge is highly beneficial for anomaly detection in connector inspection tasks. Unlike conventional PaDiM, which models feature distributions over the entire image, the proposed ROI-guided strategy explicitly directs the model toward defect-sensitive pin regions. Since missing-pin defects are inherently localized within the pin array, restricting feature extraction to these physically meaningful regions enables more discriminative anomaly representation while reducing interference from unrelated structures, illumination variations, and background noise.

Figure 5 further illustrates the performance comparison among the three ROI strategies. A clear performance trend can be observed, with detection accuracy consistently increasing from No Mask to Rectangle Mask and finally to Pin Mask. This progression highlights the importance of incorporating domain-specific structural information into unsupervised industrial anomaly detection frameworks. The results validate the effectiveness of the proposed physics-guided design and demonstrate its potential for practical deployment in automated connector inspection systems.

#### 4.2.2. Visual Analysis on Detection Heatmaps

**Figure 6:**
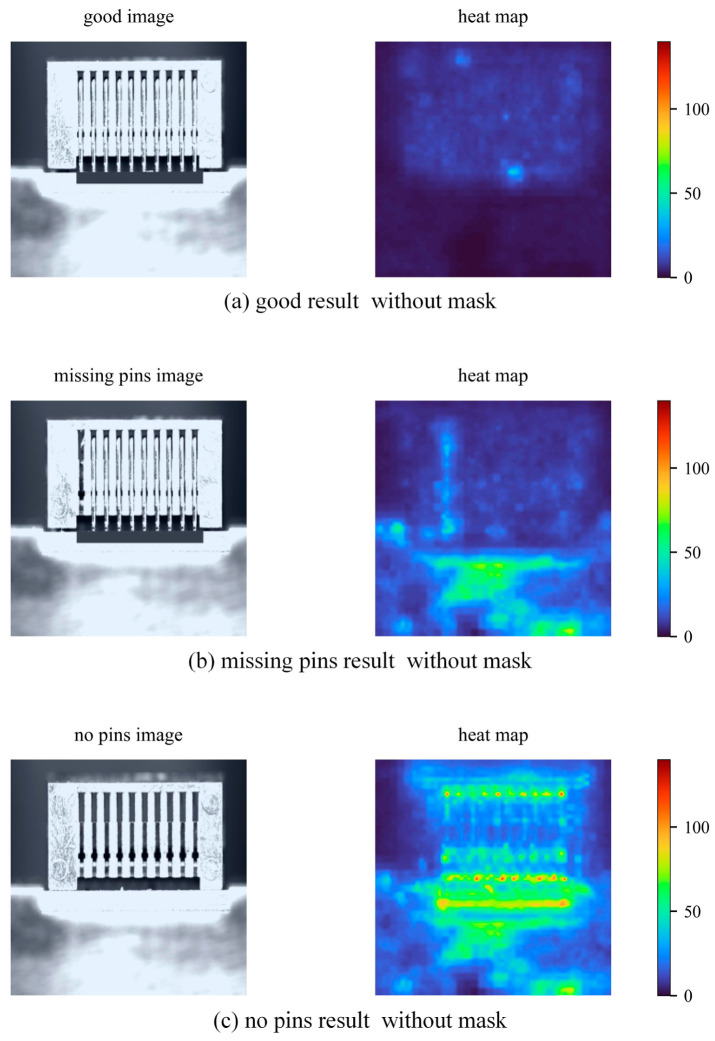
Detection results without ROI mask.

**Figure 7:**
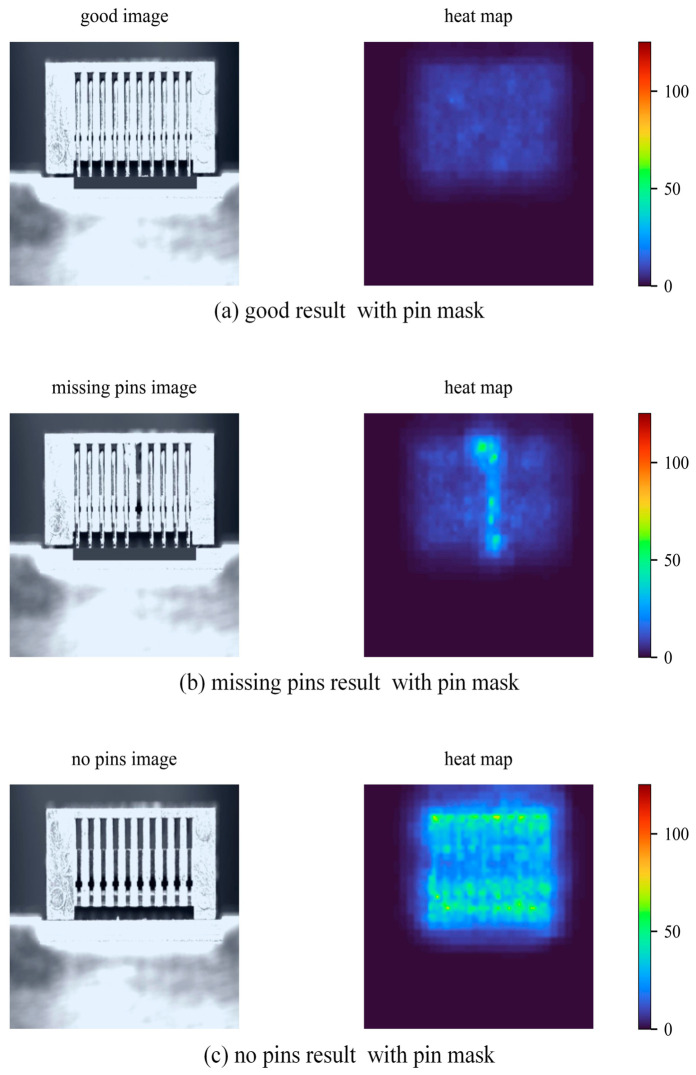
Detection results with pin mask.

To further intuitively verify the effectiveness of the proposed method, we conduct visual analysis based on pixel-level anomaly heatmaps. Figure 6 presents the detection results generated by the original PaDiM without any ROI mask, while Figure 7 shows the corresponding results of the proposed method equipped with Pin Mask. Three typical samples including good connectors, connectors with missing pins and connectors without pins are selected for comparison.

As shown in Figure 6, when working without ROI constraints, the model generates scattered abnormal responses over the whole image. For normal samples, the heatmap still produces sporadic high-value noise points on the background and connector housing areas, which are interference regions irrelevant to pin defects. For defective samples with missing pins and complete missing pins, although the model can roughly perceive abnormal areas, the anomaly response is diffused. A large number of background pixels are mistakenly judged as abnormal, leading to blurred defect localization and poor interpretability of detection results. This phenomenon is caused by the fact that the original PaDiM learns feature distributions from the entire image, and complex industrial backgrounds and uneven illumination bring extra feature disturbances.

In contrast, the heatmaps in Figure 7 obtained by the Pin Mask-based method show obvious advantages. Benefiting from the physical structural prior introduced by the pin-oriented ROI mask, the model effectively shields background interference and focuses feature modeling on the pin array region. For normal connector samples, the anomaly heatmap maintains an overall low response, with almost no false positive points, which proves that the proposed mask can eliminate the interference of non-defect areas. For defective samples, the high-value regions of the heatmap are highly concentrated on the actual defective pin positions. The model accurately highlights the local defect areas without generating widespread false alarms on the background.

The qualitative visualization results are consistent with the above quantitative AUROC evaluation. The designed Pin Mask successfully guides the model to concentrate on defect-sensitive regions. It not only improves the overall detection accuracy and stability but also realizes precise pixel-level localization of pin defects. This further proves that embedding physical structural priors via ROI masking is an effective way to optimize unsupervised anomaly detection for electrical connector pin inspection.

#### 4.2.3. Parameter Discussion

To evaluate the impact of the regularization parameter ε on the anomaly detection performance, a parameter sensitivity experiment was conducted on the evaluation set. The value of ε varied across a wide range from 0.0001 to 10, and the corresponding macro AUROC metrics are reported in Table 3.

The proposed PGPDM exhibits high robustness to parameter fluctuations. Within the interval [0.01, 0.1], the AUROC remains stable around 98%. However, extreme values degrade performance: a very small ε (<0.0001) fails to prevent numerical instability during covariance matrix inversion, dropping the AUROC to 94.75%, whereas an excessively large ε (>10) over-smooths the distinct statistical features of normal samples, lowering accuracy to 96.92%. The model achieves its peak AUROC of 98.29% at ε = 0.04, which is selected as the default setting.

## 5. Conclusions

This paper presents a physics-guided patch distribution modeling method for unsupervised detection of connector pin defects. Considering the structural characteristics of electrical connectors in industrial production, two ROI masking schemes are designed to introduce physical prior information into the deep convolutional networks, guiding the model to focus on key pin regions and filter out background interference. Extensive comparative experiments are conducted on a real industrial dataset containing normal connectors, connectors with missing pins and connectors without pins. The quantitative results show that the proposed Pin Mask obtains the highest average AUROC of 97.81% and the best stability among all strategies. Visualized heatmap results also confirm that the ROI-guided method effectively suppresses false alarms and improves the accuracy of defect localization. The proposed PGPDM framework combines the advantages of unsupervised learning and structural prior guidance, which reduces the reliance on defective sample collection and manual annotation.

However, the proposed method has currently been validated on only one specific type of connector defect inspection. Future work will explore how the advantages of this unsupervised detection framework can be further extended and applied to a broader range of production lines.

## Figures and Tables

**Table 1:** Comparison of AUROC (%) across five repeated experiments for different ROI masks.

ROI Mask	No Mask	Rectangle Mask	Pin Mask
Repeat 1	92.90%	96.28%	98.06%
Repeat 2	93.80%	96.82%	97.66%
Repeat 3	94.20%	96.25%	97.70%
Repeat 4	94.27%	96.89%	97.84%
Repeat 5	93.96%	96.68%	97.78%
Mean	93.83%	96.58%	97.81%
Std.	0.55%	0.30%	0.16%

**Table 2:** Quantitative classification performance under different ROI masks.

ROI Mask	AUROC	F1-Score	Precision	Recall
No Mask	93.83%	91.17%	95.43%	87.27%
Rectangle Mask	96.58%	92.80%	97.94%	88.18%
Pin Mask	97.81%	93.85%	98.39%	89.71%

**Table 3:** Anomaly detection performance (AUROC) under different values of ε.

ε	0.0001	0.001	0.01	0.04	0.1	1	10
AUROC	94.75%	97.53%	98.13%	98.29%	97.86%	97.67%	96.92%

## Data Availability

Data will be made available by the corresponding author upon request.
